# Sensor Systems for Detecting Dough Properties Fortified with Grape Pomace and Mealworm Powders

**DOI:** 10.3390/s20123569

**Published:** 2020-06-24

**Authors:** Martin Adamek, Anna Adamkova, Jiri Mlcek, Klara Vojackova, Oldrich Famera, Martin Buran, Veronika Hlobilova, Martina Buckova, Mojmir Baron, Jiri Sochor

**Affiliations:** 1 Department of Microelectronics, Faculty of Electrical Engineering and Communication, Brno University of Technology, Technicka 3058/10, 616 00 Brno, Czech Republic; adamek@feec.vutbr.cz (M.A.); xburan02@stud.feec.vutbr.cz (M.B.); 2 Department of Food Analysis and Chemistry, Faculty of Technology, Tomas Bata University in Zlin, Vavreckova 275, 760 01 Zlin, Czech Republic; aadamkova@utb.cz (A.A.); k_vojackova@utb.cz (K.V.); hlobilova@utb.cz (V.H.); buckova@utb.cz (M.B.); 3 Department of Food Science, Faculty of Agrobiology, Food and Natural Resources, Czech University of Life Sciences Prague, Kamycka 129, 165 00 Praha 6 - Suchdol, Czech Republic; famera@af.czu.cz; 4 Department of Viticulture and Enology, Faculty of Horticulture, Mendel University in Brno, Valticka 337, 691 44 Lednice, Czech Republic; mojmir.baron@mendelu.cz (M.B.); jiri.sochor@mendelu.cz (J.S.)

**Keywords:** flour, dough, mealworms, grape pomace, wheat flour

## Abstract

The present article dealt with the fortification of plain wheat flour by the addition of grape pomace flour and mealworm larvae powder, focusing on the mineral content and selected properties of the dough. The work also analyzed the properties of one mixture in a weight combination of 80% wheat flour, 10% grape pomace, and 10% mealworm. X-ray analysis was used to measure the mineral content of calcium, iron, copper, and zinc. The properties of the individual mixture were monitored using an experimental electronic nose and a thermodynamic sensor system during the leavening. The results showed that a combination of 50% grape pomace and 50% mealworm larvae was advantageous from the viewpoint of the favorable representation of minerals. The analyzed mixture contained a high proportion of calcium (3976.7 ± 362.9 mg·kg^−1^), iron (209.3 ± 25.7 mg·kg^−1^), and copper (65.0 ± 100.1 mg·kg^−1^) for grape pomace as well as a high proportion of zinc (277.0 ± 21.9 mg·kg^−1^) for the mealworm larvae. However, this mixture showed a small change in the heat flux response when analyzed with thermodynamic sensors (lower yeast activity and worse gas formation properties resulted from the sensor characteristic with a lower response). The 100% wheat flour had the highest response, and the second highest response was recorded for a mixture of wheat flour with 10% grape pomace and 10% mealworm larvae. This combination also often had one of the highest responses when measured with an experimental electronic nose, so this combination was considered as one of the most advantageous options for processing from the mixtures mentioned in the article.

## 1. Introduction

Nowadays, people are increasingly focusing on health and a healthy lifestyle. In the field of food, the fortification (enrichment) of food is one of the ways of improving the health condition of the population. The beginning of this dates back to the end of the 19th century, when, for example, in 1898, the Austrian professor Wagner von Jaureg proposed eliminating goiters by iodizing salt [1]. At present, due to iodine deficits in the diet of the population, other foods are fortified with iodine. This fortification is often given by legislation, for example, New Zealand introduced obligatory fortification of bread with iodine salt in 2009 [2]. The method was across-the-board, economically acceptable, technologically available, covered the entire population of a particular territory and was easily accessible to consumers. Bread and other types of bread are therefore very beneficial for enrichment with other ingredients.

Another advantage is the possibility of balancing the nutritional value of a given bakery product, for example, by means of components with the presence of biologically active substances. Grape pomace is an example. It is further used in the production of alcohol, as a basic raw material to produce grape oil, and after drying and grinding as grape pomace flour (GPF). Grape pomace is rich in polyphenols, especially flavonoids such as gallic acid, catechin, and epicatechin [3], which can have a positive impact on human health [4,5,6]. Furthermore, in grape pomace, except for the phenolic compounds with antioxidant activity, other antioxidants neutralize free radicals and can thus serve as prevention against civilization diseases such as type 2 diabetes, obesity, and cancer [7]. Non-traditional commodities can also be used to enrich bakery products such as edible insects [8]. Edible insects are foods that are consumed mainly in Asia, Africa, and Latin America [9]. In European countries, for example, in Italy or in France, Casu Marzu cheese [10] consists of proteins, minerals, and n-3 and n-6 fatty acids.

In mealworm larvae (*Tenebrio molitor*), which is one of the most studied species of edible insects, the crude protein content in the dry matter is reported to be 45.1% to 63.0% [11,12,13]. The fat content of mealworm larvae is reported to range from 18.9% to 38.3% [11,12,13,14]. The content of polyphenolic fatty acids in edible insects makes up 70% of the total fat content [15]. The content of individual nutrients depends mainly on the breeding temperature and feed.

In the case of grape pomace, fiber predominates in the composition. Zalikarenab et al. [16] stated that the content of neutral detergent fiber for white grape pomace was 51.5% and was 58.0% in red grape pomace. In contrast, the crude protein content was determined to be 12.2% and 8.9%. Similarly, fat is present in small amounts (5.0% and 7.1%). Mohamed Ahmedet al. [17] reports crude fiber in the range of 36.8–51.8%, crude protein in the range of 9.3–14.4%, and crude oil in the range of 6.4–10.5% for various wines. This allows complementarity with flour from mealworm larvae. Another advantage is that the insect itself is tasteless [18]. It is obtained only after mixing with other ingredients, and therefore the edible insects can be used in many different foods.

This issue has been studied particularly in the field of bakery products, where it is possible to combine the advantages that this commodity offers in the way of increasing nutritional value, flavor taking, has a powder form allowing easy implementation, and invisibility for the consumer [19,20]. Aguilar-Miranda et al. [21] examined tortilla dough with the addition of mealworm powder, which increased the amount of essential and semi-essential amino acids. The results confirmed the improved nutritional value of flour for the production of bread fortified by insects as reported in the study by González et al. [22]. Edible insects are an important source of proteins, amino acids, fats, chitin, and some minerals such as zinc. The amount of some minerals in mealworm larvae and superworm was investigated by Mlček et al. [23] using a simple hand held X-ray analyzer. Using classical analytical methods, the content of elements has been examined, for example, Wu et al. [24], determined the elements using a high-resolution continuum source atomic absorption spectrometer. In the case of mealworm, they found Ca values of 319.6 mg·kg^−1^, Fe of 184.2 mg·kg^−1^, Cu of 20.2 mg·kg^−1^, and Zn of 98.6 mg·kg^−1^.

On the other hand, grape pomace flour contains other minerals, polyphenols, and antioxidants. Values of 4.4 mg·kg^−1^ calcium, 180.1 mg·kg^−1^ iron, and 9.8 mg·kg^−1^ zinc were determined by atomic absorption spectrophotometry by Sousa et al. [25]. However, Mohamed Ahmed et al. [17] reported calcium values in the range of 2560–9610 mg·kg^−1^. By adding a suitable combination of these substances (the synergism between grape pomace flour and mealworm larvae powder) to ordinary baker’s flour, its nutritional value can be improved.

Monitoring the properties of flour and dough using fast, undemanding, and ideally cheap methods is important, especially in small and domestic productions. A modern non-contact method can be an electronic nose, which can monitor the gases produced during fermentation [26]. Another non-traditional method can be the use of thermodynamic balance sensors that have already been tested in the fermentation of dairy products [27].

Scientific Hypothesis

**Hypothesis** **1.** *Addition of grape pomace flour and/or mealworm larvae powder to common wheat flour will increase the concentration of mineral elements.*

**Hypothesis** **2.** *The experimental electronic nose will record changes in the gas concentration of the dough leavening from a mixture of common wheat flour and flour with the addition of grape pomace flour and/or mealworm larvae powder.*

**Hypothesis** **3.** *The thermodynamic sensor will detect heat changes in the leavening of dough mixtures of common wheat flour and flour with the addition of grape pomace flour and/or mealworm larvae powder.*

The aim of the present work was to determine and compare the representation of mineral elements in common commercial wheat flour and the flour with the addition of grape pomace flour and/or mealworm larvae powder using a hand-held X-ray analyzer.

Another aim of the work was to investigate the possibilities of experimental electronic noses and simple devices with thermodynamic sensors to monitor dough leavening from the above-mentioned mixtures to find possible differences.

## 2. Materials and Methods

### 2.1. Materials

The basic material was commercial flour purchased in the supermarket as a commonly available material like plain wheat flour “Babiccina volba” (flour type 530, ash content up to 0.60%), GoodMills Czech Republic Inc., Prague, The Czech Republic, with nutritional values per 100 g: energy was 1461 kJ/345 kcal; fats were 1.7 g, of which saturated fatty acids were 0.2 g; carbohydrates were 69 g, of which sugar was 2.0 g, fiber was 3.1 g, proteins were 12 g, and salt was <0.01 g.

Two types of materials were used for fortification: grape pomace flour and mealworm powder. Table 1 shows the labelling and composition of the individual flour mixtures. Grape pomace flour was obtained from viticulture Ludwig, vineyard track “Zbavce”, village Nemcicky, region Velkopavlovicka, area Morava. Material for the grape pomace homogenates was acquired from grapes of the Riesling variety, which was manually harvested in October 2018. Next, the grapes were processed using a time-driven horizontal press. Drying and grinding were carried out at 70 °C.

Mealworm powder (mealworm flour) was obtained from the larvae of the mealworm (*Tenebrio molitor*). Larvae in the last and penultimate developmental stages (full length of the body just before the pupae) were used. Larvae were purchased from Radek Fryzelka Company, Brno, Czech Republic, and were reared at Tomas Bata University in Zlin, Czech Republic for six weeks. The larvae were taken from the breed, left to starve for 48 h, killed in boiling water at 105 °C, and immediately dried at 105 °C. The samples prepared in this way were homogenized and stored in a refrigerator at 4–7 °C until analysis.

### 2.2. Determination of Minerals by X-ray Analysis

**Figure 1:**
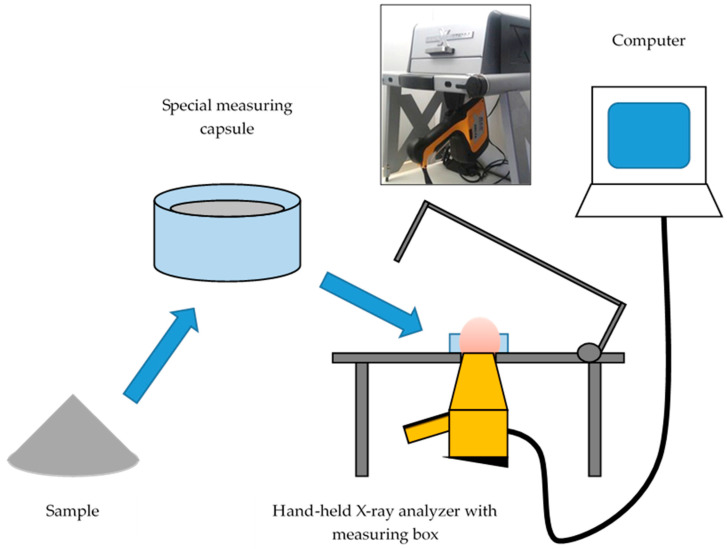
The schematic diagram of the measurement of the detection of elements using a simple handheld X-ray analyzer Innov-X DELTA (Innov-X Systems, INC., Woburn, MA, USA).

Elements were determined using X-ray fluorescence (XRF) analysis. Each sample was put in a special measuring capsule (cylindrical shape, diameter approx. 25 mm, height approx. 20 mm, full capsule (approx. 5 g)), and then placed in the measuring box for analysis, Figure 1. Subsequently, they were measured using a simple handheld X-ray analyzer Innov-X DELTA (Innov-X Systems, INC., Woburn, MA, USA). The analysis was triggered and recorded using the control program DELTA Premium PC Software (Innov-X Systems, INC, Woburn, MA, USA) [23]. The measurement was performed automatically where the mode was set to RoHS/WEEE (Restriction of Hazardous Substances Directive / Waste Electrical and Electronic Equipment Directive). The maximum measurement time was set to 90 s. Samples were measured three times and were analyzed at a room temperature of 23 ± 2 °C.

### 2.3. Monitoring Using Experimental Electronic Nose

Dough leavening was monitored using an experimental electronic nose. The basis of construction is described in Adámek et al. [28]. The electronic nose was expanded to include temperature and humidity measurements using the commercial ASAIR AM 2302 (DHT22) sensor (Guangzhou Aosong Electronics Co., Ltd., Guangzhou, China) and SGP30 sensor (Sensirion AG, Staefa ZH, Switzerland) for total volatile organic compound (TVOC) and H_2_-based CO_2_ equivalent. Both quantities had a digital output with a numerical value of the measured quantity and were calculated from the basic signals for ethanol and H_2_. Replacing the MQ-6 sensor with the MQ-135 sensor (Zhengzhou Winsen Electronics Technology Co., Ltd., Zhengzhou, China) was the last modification in the sensor part of the electronic nose. Generally, the MQ-135 sensor is sensitive to ammonia gas, toluene, hydrogen, and smoke in the range of 10–1000 ppm. However, the experiment assumed monitoring the relative change in ammonia gas concentration. Furthermore, the MQ-3 sensor, which is very sensitive to alcohol (25–500 ppm), and the MQ-8 sensor designed for hydrogen detection (100–1000 ppm) were used.

Since this device was only to monitor the process and accurate measurement of the absolute values of individual gas concentrations in the measured odor was not expected, individual gas concentration signals in voltage (MQ-135, MQ-3, and MQ-8 sensors) were converted to relative scales using the internal 10-bit A/D converter of the microcontroller to digital values d [-] (from voltage level 0–5 V to digital level 0–1023) [28].

**Figure 2:**
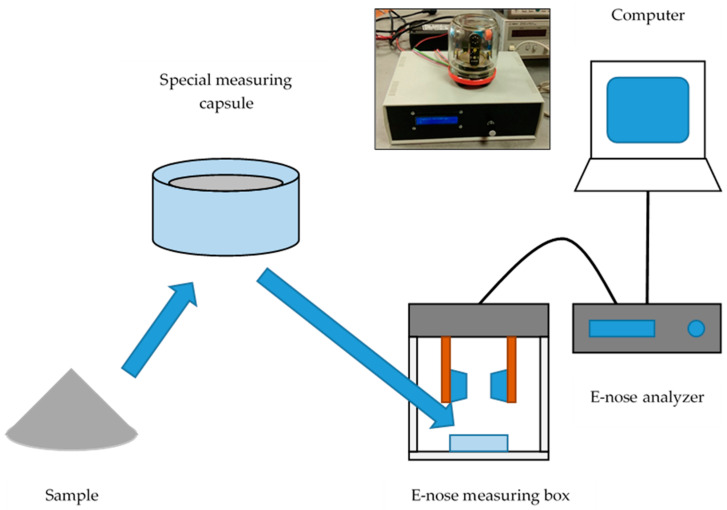
The schematic diagram of measuring dough leavening using a simple experimental electronic nose.

The sample (flour mix 5 g, distilled water 5 mL, and dried yeast 0.3 g) was put in a plastic measuring container (cylindrical shape, diameter approx. 40 mm, height approx. 30 mm), mixed, and then placed in the measuring glass box for analysis, as shown in Figure 2. A sample was inserted in the box at t = 600 s and was monitored for 2400 s minimally. Each sample was analyzed at a temperature of 35 ± 3 °C with two repetitions.

### 2.4. Monitoring by Thermodynamic Sensors

Fermentation of various mixtures of fortified flours was analyzed using the thermodynamic sensors (TDS) described in Adámek et al. [27]. The principle of these thermodynamic sensors is based on the measurement of energy, which is supplied to circuit the temperature setting and equilibration of the temperature element as ambient. An amplifier and converter to the electrical signal are very often integrated with the sensor element. Therefore, the system is very easily connected to other measuring systems [27].

**Figure 3:**
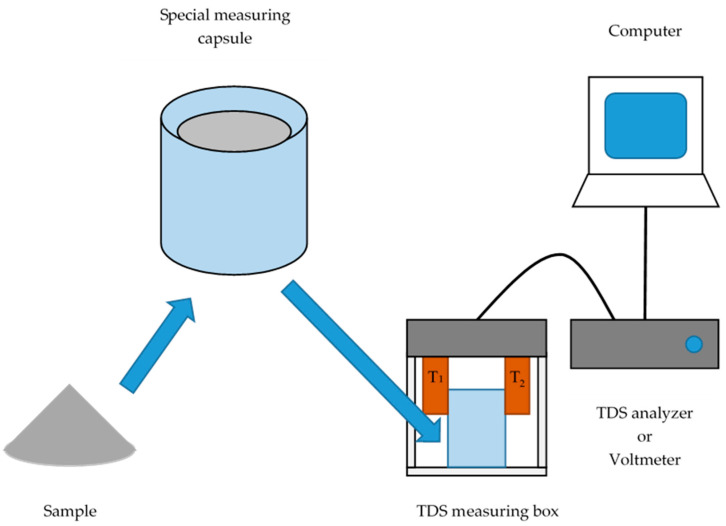
The schematic diagram of the fermentation measurement of various mixtures using thermodynamic sensors.

In the circuit, two measuring elements T1 and T2 are located as shown in Figure 3. If the temperature effects affect the temperature of both elements in the same way (e.g., ambient interference), the output signal of the circuit does not change. If the temperature effect acts on only one element or the temperature flow between the element changes, the voltage of the circuit changes, which is further amplified, measured, and processed by a computer.

The sample (flour mix 5 g, distilled water 5 mL, and dried yeast 0.3 g) was put in a plastic measuring cup, mixed, and then placed in the special measuring glass box for analysis (Figure 3). A sample was inserted in the box at t = 300 s and was minimally monitored for the next 2400 s. Each sample was analyzed at a temperature of 23 ± 2 °C in two repetitions. The supply voltage of the experimental equipment was 20 V and therefore, the possible output voltage of the device was in the range of 1–19 V.

### 2.5. Statistical Analysis

The data obtained for the determination of selected elements were evaluated using Microsoft Excel 2013 (Microsoft Corporation, Redmond, Wash., USA). Results were expressed as the mean (M) ± standard deviation (SD).

Although two curves were determined for each type of mixture for the electronic nose and TDS monitoring, the curves were set to the same starting position, and the resulting average curve was calculated from them. Therefore, this is preliminary information.

## 3. Results and Discussion

In the first part of the work, the representative elements (Ca, Fe, Cu, and Zn) were analyzed in wheat flour, grape pomace flour, mealworm flour, and their mixtures. The results obtained are shown in Table 2.

In plain wheat flour, none of the monitored elements except zinc (22.3 ± 2.5 mg·kg^−1^) was detected by the handheld X-ray analyzer. The Czech Nutritional Database [29] lists the zinc content of 7.3 mg·kg^−1^ for the same type of flour (type 530). This is consistent with Ertl and Goessler [30], who compared the elements in different flours. In their case, overall, the range was from 3.8 mg·kg^−1^ to 50 mg·kg^−1^. A similar result was reported by Xing et al. [31], where the amount of zinc in whole grain flour was in the range of 24.7–42.7 mg·kg^−1^. The zinc content in the measured plain wheat flour was therefore similar to that of other authors. The detected zinc content of grape pomace flour was twice as high as that of wheat flour (44.3 ± 24.8 mg·kg^−1^), but even that was only 11% of the value determined for mealworm flour. However, the other monitored values of elements were higher in grape pomace flour which had, in particular, a high proportion of calcium and iron.

A high content of elements with an even representation of all monitored elements including zinc was found in a sample containing 50% grape flour and 50%mealworm flour. However, this mixture did not show suitable gas-forming properties in further tests. A mixture of 80% wheat flour, 10% grape flour, and 10% mealworm flour showed more advantageous properties from the investigated samples. This mixture showed an increase in all monitored minerals except calcium. However, it was assumed that the concentration of this element also increased, but this could not be recorded due to the detection limit for calcium in this analyzer.

In the FoodData Central (USDA) database, calcium content is reported most frequently in the range of 150–400 mg·kg^−1^ and the Czech Nutritional Database [29] reports that flour type 530 shows a calcium content of 210 mg·kg^−1^. Pande et al. [32] performed measurements on wholemeal flour Atta, where Ca values reached 239 ± 0.2 mg·kg^−1^ as opposed to wheat flour where calcium was not detected. Ertl and Goessler [30] determined calcium values of 190 ± 30 mg·kg^−1^ for wheat flour (type 480), 190 ± 50 mg·kg^−1^ for wheat flour (type 700), and 390 ± 80 mg·kg^−1^ for whole wheat flour. In contrast, Sousa et al. [25] determined the calcium content of 4400 mg·kg^−1^ or Bennemann et al. [33] from 1580 mg·kg^−1^ to 4295 mg·kg^−1^ in grape pomace flour. This was comparable to the measured sample of grape pomace flour that was more than seven times higher than the calcium content of mealworm flour and may be up to 30 times higher than that of commercial plain wheat flour.

Except for wheat flour, iron values were recorded in all other flours. The Czech Nutritional Database [29] lists an iron content of 10 mg·kg^−1^ in wheat flour type 530 and in the FoodData Central (USDA) database, the iron content is most often in the range of 15–50 mg·kg^−1^. Iron content in 100% plain wheat flour type 530 (10 mg·kg^−1^ taken from the Czech Nutritional Database) was more than seven times lower than in mealworm flour and more than 25 times lower in grape pomace flour. Ertl and Goessler [30] found iron levels of 7.1 ± 3.2 mg·kg^−1^ for wheat flour (type 480), 11 ± 2 mg·kg^−1^ for wheat flour (type 700) and 34 ± 3 mg·kg^−1^ for whole grain wheat flour. Therefore, it can be generally stated that wheat flour containing 10% grape pomace flour reached similar values of iron to that of rye flour (20 ± 1 mg·kg^−1^ for rye flour, 23 ± 1 mg·kg^−1^ for rye whole grain flour) [30]. Wheat flour with a 10% addition of mealworm flour reached on average the same values as spelt flour (14 ± 3 mg·kg^−1^) and was slightly higher than that of wheat flour (type 700) [30].

The value of copper in the sample of 100% plain wheat flour was below the detection limit. In the Czech and American Nutritional Databases, the copper content of the same type of flour was not found. Ertl and Goessler [30] found copper values of 1.4 ± 0.2 mg·kg^−1^ for wheat flour (type 480), 1.8 ± 0.2 mg·kg^−1^ for wheat flour (type 700), and 4.4 ± 0.6 mg·kg^−1^ for wholemeal wheat flour. Copper values for 100% mealworm flour and 100% grape pomace flour were at least 10–15 times higher than that for wheat flour.

**Figure 4:**
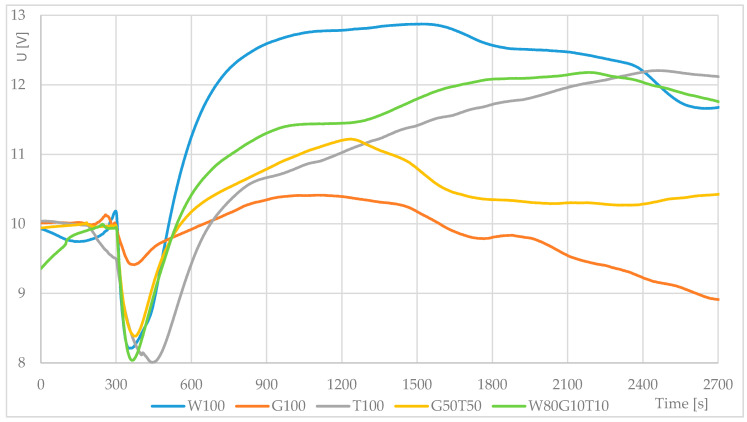
Fermentation process—average thermodynamic sensor response for different flour mixtures (W—wheat flour, G—grape pomace flour, T—mealworm flour). Sample inserted at t = 300 s.

This was followed by monitoring the leavening using a thermodynamic sensor. The resulting average recorded curves are shown in Figure 4. The results showed a rapid rise and a high temperature change in 100% wheat flour. Conversely, 100% grape flour reflected a very small change in heat flow (and hence the heat emitted or consumed). Although 100% mealworm flour produced a more pronounced drop in the curve at the start of the measurement and a slower start, it eventually reached the same value as a blend with 80% wheat flour. Although this mixture had a steeper slope of the monitored curve (changes in temperature flux) than 100% mealworm flour, it was milder in other parts. For the last of the measured mixtures (G50T50), the pattern of growth was similar to that of a sample of 100% mealworm flour, but at a certain phase, there was a decrease and stabilization. In general, other available literature examines ambient temperature such as Hirasawa and Yokoigawa [34], where the leavening measurement was performed at 30 °C for five hours.

**Figure 5:**
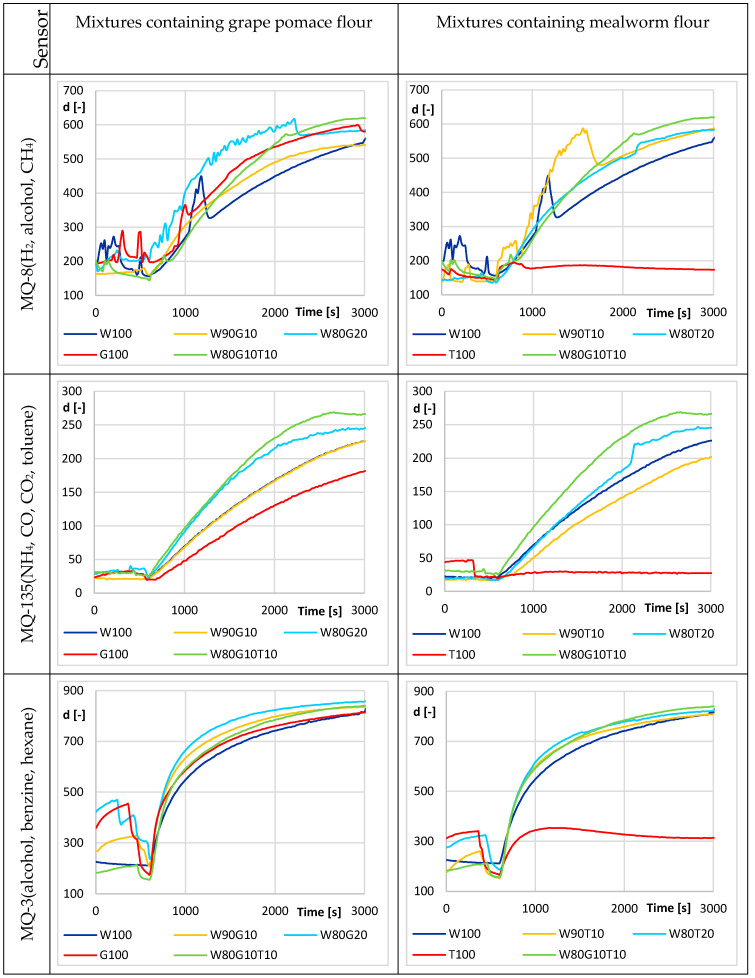
Fermentation process—average responses of the analogy electro-chemical sensors for different flour mixture (W—wheat flour, G—grape pomace flour, T—mealworm flour). Mixture sample inserted at t = 600 s.

Simultaneously with the fermentation measurement using thermodynamic sensors, the changes in the gases produced by the dough were monitored using an experimental electronic nose. The MQ-8, MQ-135, and MQ-3 sensors (Figure 5) showed little or almost no gas evolution in 100% mealworm flour. Therefore, the monitored gases did not change, and the dough did not leaven. Although it was not possible to evaluate other curves as statistically different, initial graphs suggested that adding 20% mealworm flour to wheat flour resulted in a greater evolution of monitored gases over 100% wheat flour. It was believed that the presence of a substance contained in this flour during yeast activity increased the gas-forming capacity. The situation was similar for grape pomace flour additions. The small amount of grape pomace flour (10%, 20%) added to the wheat flour again increased the volume of the evolved monitored gases. The MQ-135 sensor, which responded most to NH_4_, significantly decreased the measured curves below the signal level of 100% wheat flour, in the case of 100% grape pomace flour and 100% mealworm flour. In general, this was the case with all sensors and a 100% sample of mealworm flour. It was assumed that yeasts did not have a basic substrate for its activity in this sample. According to Figure 5, the W80G10T10 mixture appeared to have the highest evolution of the monitored gases, and thus the mixture with the most favorable gas-generating capability during leavening.

**Figure 6:**
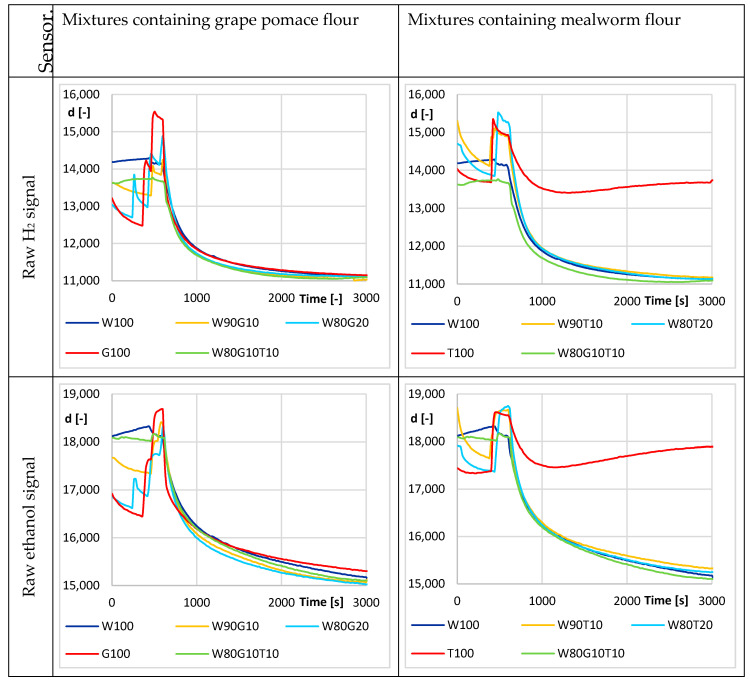
Fermentation process—average sensor response SGP-30 for different flour mixtures (W—wheat flour, G—grape pomace flour, T—mealworm flour). Mixed sample inserted at t = 600 s.

In the combined SGP30 sensor, mainly the raw signals for H_2_ and ethanol were used to monitor, but they were mirrored against the previous sensors (Figure 6). Again, both signals showed only small variations in the values of 100% mealworm flour against those of the other samples. No difference was found between the other samples.

**Figure 7:**
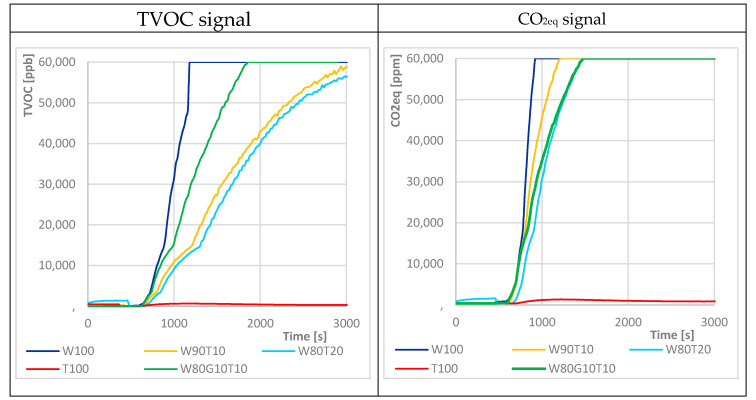
Fermentation process—examples of sensor response SGP-30 for TVOC and CO_2eq_ signal and for various contents of mealworm flour (W—wheat flour, G—grape pomace flour, T—mealworm flour). Mixed sample inserted at t = 600 s.

The raw signals for H_2_ and ethanol were then used in the SGP30 sensor for signal processing and calculation of the TVOC and CO_2eq_ signals. Due to the frequent overload of the sensor range, it was not possible to monitor these gases throughout the dough leavening. Nevertheless, the response examples (Figure 7) might have indicated a certain trend in which the concentration of individual gases decreased with increasing content of mealworm flour. The increase in signal in W80G10T10, which in previous cases appeared to be the mixture with the most favorable gas-generating capability, was smaller than that of the W100. This might have been due to the formation of more ammonia at the expense of CO_2_, to which the SGP-30 and MQ-135 sensors were otherwise sensitive. However, these results need to be confirmed by further measurements.

In practice, gas evolution can be controlled by a reofermentometer using a gas leak curve from the dough (ANONYM.Rheo). With this characteristic, it is possible to determine the volume of gas retained in the dough and the volume of gas released from the dough during leavening. Measurement of this characteristic can take several hours and individual gases are not recognized.

In the study by González et al. [22] comparing doughs with the addition of flour from selected insect species, it was reported that these additions affected the rheological properties of the dough. According to the analysis of the rheological properties mentioned in the article, the dough with the addition of mealworm larvae is similar to wheat dough. Roncoliniet al. [8] extended this information even further with the measured information and provided a comparison of the rheological properties of the dough only from wheat flour and flour containing different amounts of added mealworm flour, namely 5% and 10%. The addition of mealworm powder improved volume and softness.

## 4. Conclusions

The first part of the present work was focused on the analysis of elements in wheat flour with the addition of grape pomace flour and mealworm flour. Mealworm flour contained the highest proportion of zinc in comparison to the other flours analyzed. Calcium, iron, and copper were most represented in grape pomace flour. By combining these flours, commodities with a favorable representation of these elements can be achieved.

In the next part of the work, the leavening of the doughs of said mixtures was measured using thermodynamic sensors. The sensors recorded the highest changes in the thermal properties of common wheat flour. From the point of view of leavening and the representation of minerals, the combination of common flour with the fortification of grape pomace flour and mealworm flour appeared to be the most suitable variant up to 20% of the total content of the addition. The 100% grape pomace flour showed only a very small change in heat flow. A larger change was recorded for the G50T50 mixture, but it soon decreased almost to the start value, similar to the G100 mixture. Although the T100 mixture had a slower increase at the beginning, it was equal to the maximum value of the W80G10T10 mixture.

For the electronic nose, only a small response (in some cases no response) was found for the T100 mixture with all the sensors used. With a higher proportion of wheat flour, the response value was significantly higher. The same was true for the grape pomace and the TVOC sensor. For other sensors and G100 mixture measurements, the signal was comparable or only slightly lower. Due to the smaller signals in the W100 sample, it was possible to infer a maximum at a certain value of the addition of grape pomace flour or mealworm flour to wheat flour. However, this statement still needs to be tested on more types of mixtures and a larger number of samples.

The work shows that in order to maintain good leavening dough properties, only a limited amount of mealworm flour and grape pomace flour can be added, but this has been shown to increase the element content of the bakery product.

## Figures and Tables

**Table 1:** The compositions of the individual mixtures used for analysis and monitoring.

Mixture	Material		
	W (%)	G (%)	T (%)
W100	100	0	0
W90G10	90	10	0
W80G20	80	20	0
G100	0	100	0
W90T10	90	0	10
W80T20	80	0	20
T100	0	0	100
G50T50	0	50	50
W80G10T10	80	10	10

Note: W—wheat flour, G—grape pomace flour, T—mealworm larvae powder.

**Table 2:** Representation of elements in wheat flour, grape pomace flour, and mealworm larvae Powder.

Sample	Element			
	Ca (mg·kg^−1^ ± SD)	Fe (mg·kg^−1^ ± SD)	Cu (mg·kg^−1^ ± SD)	Zn (mg·kg^−1^ ± SD)
W100	<LOD	<LOD	<LOD	22.3 ± 2.5
W90G10	<LOD	24.3 ± 12.7	<LOD	24.3 ± 3.2
W80G20	<LOD	33.7 ± 11.7	14.0 ± 3.0	26.7 ± 4.0
G100	4985.3 ± 991.5	261.3 ± 124.6	80.0 ± 6.1	44.3 ± 24.8
W90T10	<LOD	14.3 ± 0.6	<LOD	61.3 ± 3.5
W80T20	<LOD	33.3 ± 7.4	15.0 ± 3.5	131.7 ± 21.5
T100	685.7 ± 30.4	73.7 ± 5.1	53.0 ± 1.0	395.0 ± 10.4
G50T50	3976.7 ± 362.9	209.3 ± 25.7	65.0 ± 10.1	277.0 ± 21.9
W80G10T10	<LOD	26.3 ± 6.4	18.0 ± 4.4	76.0 ± 4.6

Note: W—wheat flour, G—grape pomace flour, T—powder of mealworm larvae, SD—standard deviation.
